# The overmethylated genes in *Helicobacter pylori*-infected gastric mucosa are demethylated in gastric cancers

**DOI:** 10.1186/1471-230X-10-137

**Published:** 2010-11-20

**Authors:** Seung-Jin Hong, Jung-Hwan Oh, Eun-Jung Jeon, Ki-Ouk Min, Moo-Il Kang, Sang-Wook Choi, Mun-Gan Rhyu

**Affiliations:** 1 Department of Microbiology, College of Medicine, The Catholic University of Korea, Seoul, Korea; 2 Department of Internal Medicine, College of Medicine, The Catholic University of Korea, Seoul, Korea; 3 Department of Clinical Pathology, College of Medicine, The Catholic University of Korea, Seoul, Korea

## Abstract

**Background:**

The transitional-CpG sites between weakly methylated genes and densely methylated retroelements are overmethylated in the gastric mucosa infected with *Helicobacter pylori *(*H. pylori*) and they are undermethylated in the gastric cancers depending on the level of loss of heterozygosity (LOH) events. This study delineated the transitional-CpG methylation patterns of CpG-island-containing and -lacking genes in view of the retroelements.

**Methods:**

The transitional-CpG sites of eight CpG-island-containing genes and six CpG-island-lacking genes were semi-quantitatively examined by performing radioisotope-labelling methylation-specific PCR under stringent conditions. The level of LOH in the gastric cancers was estimated using the 40 microsatellite markers on eight cancer-associated chromosomes. Each gene was scored as overmethylated or undermethylated based on an intermediate level of transitional-CpG methylation common in the *H. pylori*-negative gastric mucosa.

**Results:**

The eight CpG-island genes examined were overmethylated depending on the proximity to the nearest retroelement in the *H. pylori*-positive gastric mucosa. The six CpG-island-lacking genes were similarly methylated in the *H. pylori*-positive and -negative gastric mucosa. In the gastric cancers, long transitional-CpG segments of the CpG-island genes distant from the retroelements remained overmethylated, whereas the overmethylation of short transitional-CpG segments close to the retroelements was not significant. Both the CpG-island-containing and -lacking genes tended to be decreasingly methylated in a LOH-level-dependent manner.

**Conclusions:**

The overmethylated genes under the influence of retroelement methylation in the *H. pylori*-infected stomach are demethylated in the gastric cancers influenced by LOH.

## Background

A mouse model infected with *Helicobacter pylori *(*H. pylori*) has illustrated that bone marrow stem cells migrate to the gastric mucosa and then they differentiate into gastric epithelial cells [1]. According to a self-organization model, highly expressed master genes can form a transcription hub to coordinate the expression of many other genes [2]. A dosage compensation mechanism proposes that there is an inverse correlation between the housekeeping genes containing CpG-islands and the tissue-specific genes lacking CpG-islands, which both share a limited amount of nuclear proteins in the nuclear space [3,4]. Both the self-organization and dosage compensation models highlight that the epigenetic co-regulation of highly active stomach-specific genes and weakly active housekeeping genes facilitates the trans-differentiation of marrow-derived cells in the stomach.

Individuals infected with *H. pylori *frequently undergo a series of gastric mucosal changes including precancerous and cancerous lesions [5]. Although CpG-island overmethylation that may result in the inactivation of cancer-associated genes is common in the *H. pylori*-infected gastric mucosa, an association between the overmethylation and expression of individual CpG-island genes is ambiguous [6]. Gastric precancerous and cancerous lesions have shown CpG-island overmethylation as well as genome-wide undermethylation, but the two distinct methylation changes showed no cooperation for the sequential evolution of precancer and cancer [7]. In addition, the highly expressed stomach-specific genes playing a master role in the co-regulation of numerous genes have demonstrated a few methylation changes in the gastric cancers [4,8]. With respect to coordinate gene expression, it is necessary to delineate if the stomach-specific genes and the housekeeping genes undergo concurrent over- and under-methylation changes.

The retroelements are self-replicating parasitic DNAs that occupy half the human genome [9]. The host genome suppresses the hazardous mutation effect of the parasitic retroelements via a DNA methylation-dependent mechanism [10]. The transitional area between the weakly methylated genes and the densely methylated retroelements, such as the CpG-island margin and the transcription start site lacking CpG-islands, is methylated to various degrees in a tissue-type-dependent manner [4,11,12]. The methylation of transitional-CpG sites changes dynamically in response to the trans-differentiation of bone marrow stromal cells and the loss-of-heterozygosity (LOH) events in gastric cancers [12-15]. Another previous study also has described that the "CpG-island shore" is related with the regulation of cell differentiation and carcinogenesis [16]. Interestingly, many transitional-CpG sites and gene-adjacent retroelements are simultaneously over- or undermethylated in a given tissue [11,17], indicating that the concurrent methylation changes in numerous genes are under the influence of retroelement methylation. Meanwhile, most CpG-rich islands are weakly methylated or unmethylated in most tissue types, and the CpG-rich sites are not suitable for the analysis of dynamic methylation adjacent to gene-control regions [12,13,15,18]. Therefore, the transitional-CpG sites, rather than CpG-rich islands, are likely to serve as pivot-methylation positions that reflect the concurrent methylation patterns associated with *H. pylori *infection and the evolution of cancer.

This study investigated the transitional-CpG methylation patterns of the CpG-island genes and the stomach-specific genes lacking CpG-islands in *H. pylori*-infected gastric mucosa and gastric cancers. The variable methylation of transitional-CpG sites was semi-quantitatively estimated under stringent methylation-specific PCR (MSP) conditions that produced clear DNA bands [4,8]. An increase or decrease in the transitional-CpG methylation of each gene was determined based on an intermediate methylation in the *H. pylori*-negative gastric mucosa.

## Methods

### Collection of tissue samples

Non-cancerous tissue samples were collected from the consecutive outpatients who underwent gastric endoscopy from April 2008 to October 2009 at St. Paul's Hospital, The Catholic University of Korea. During endoscopic examination, two adjacent tissue samples were obtained from the stomach antrum and body portion, respectively. One biopsy specimen was fixed with formalin for the histologic examination and the other biopsy specimen was used for DNA extraction. The pathologist confirmed a gastric epithelial cell content of more than 80% purity in the biopsy tissues. *H. pylori *infection was examined using the Warthin-Starry silver impregnation method. This study included 50 antrum and body pairs in *H. pylori*-negative cases with a mean age of 53.2, and 50 antrum and body pairs in *H. pylori*-positive cases with a mean age of 55.6. There were 25 males and 25 females in the *H. pylori*-negative cases and 26 males and 24 females in the *H. pylori*-positive cases. The gastric cancer tissues were obtained from the pathologic specimens of 48 male and 22 female patients (mean age: 63.7), who underwent surgical resection between March 2005 and December 2008 at St. Paul's Hospital, The Catholic University of Korea. The clinicopathologic tumor stage was determined according to the Tumor-Node-Metastasis (TNM) criteria [19]. All the subjects provided their written informed consent and this study was approved by the institutional review board.

### Tissue preparation and bisulfite modification of DNA

The fresh biopsy specimens were used for feasible semi-quantitative MSP analysis, because formalin-fixed tissue DNAs tend to be poorly amplified after bisulfite modification [8]. For the microsatellite analysis, the DNA was extracted from the formalin-fixed paraffin-embedded tumor tissues as described previously [20,21]. The tumor specimens were microdissected using a surgical scalpel under a stereomicroscope. All of the microdissected cancer tissues contained a cancer cell content of more than 80%. Using DNA extraction buffer (0.5% Tween-20, 1 mM EDTA pH 8.0, 50 mM Tris pH 8.0, 0.5 mg/mL proteinase K), the biopsy specimens and microdissected tissues were digested at 37°C for 24 hr. Approximately 1,000 cells were incubated with 20 μL of the DNA extraction buffer after which a DNA isolation kit (A1120, Promega, Madison, WI, USA) was used to extract the genomic DNA according to the manufacturer's instructions.

Tissue DNA was modified using sodium bisulfite as described elsewhere [8,11-13]. Briefly, 1 μg genomic DNA was treated with 10 μL of 3 M NaOH for 15 min at 37°C. Then the denatured DNA was mixed with 1.04 mL of 2.3 M sodium bisulfite and 60 μL of 10 mM hydroquinone and this was warmed at 50°C for 12 hrs. The bisulfite treated DNA, which was purified using Wizard DNA purification resin (Promega, Madison, WI, USA), was desulfonated with 3 M NaOH and precipitated with ethanol, and then was dissolved in 35 μL of 5 mM Tris buffer (pH 8.0).

### Radioisotope-labelling semiquantitative methylation analysis

**Figure 1:**
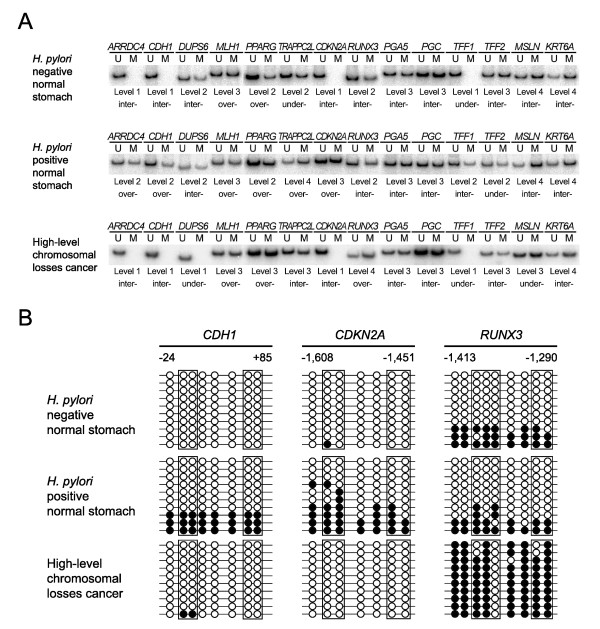
**The semi-quantitative methylation analysis (A) and the cloning and sequencing (B) in the transitional-CpG sites**. The methylation of transitional-CpG sites was evaluated by performing a semi-quantitative methylation analysis (A) and it was verified with cloning and sequencing of the common primer (B). (A) A total of 14 transitional-CpG sites were examined in the *H. pylori*-negative and *H. pylori*-positive normal gastric mucosa and cancer lesion of gastric cancer patients. The methylation density was calculated according to the proportion of the methylation (M) band intensity against the total unmethylation (U) and methylation band intensity. The methylation density of each CpG site was classified into 5 levels with 20%-methylation increment. The methylation status of each gene was categorized as undermethylation (under-) or overmethylation (over-) based on an intermediate methylation level (inter-) of the *H. pylori*-negative normal gastric mucosa. (B) Representative results of cloning and sequencing of common PCR. The transitional-CpG sites of the *CDH1*, *CDKN2A *and *RUNX3 *genes were analyzed by cloning and sequencing the common PCR product. The genomic DNAs represented in Figure 1A were used to compare the MSP results with the sequencing results. The open and closed circles indicate unmethylated and methylated cytosine residues, respectively. The rectangular boxes indicate the MSP primer sites.

Comparative analysis of the microarray and SAGE (Serial Analysis of Gene Expression) data has found that the number of transcripts counted in the SAGE data accurately represents a great difference in the gene activity between the stomach-specific genes and housekeeping genes [4]. The transitional-CpG sites of the stomach-specific genes (*PGA5 *and *PGC*) [22], the mucosa-healing genes (*TFF1 *and *TFF2*) [23], the cancer-related genes (*CDH1*, *MLH1*, *PPARG*, *CDKN2A *and *RUNX3*) [15,24-27], and the non-gastric genes (*ARRDC4*, *DUSP6*, *TRAPPC2L *, *MSLN *and *KRT6A*) [28-32] were selected (Table 1). The MSP sites, sequences, and conditions are shown in Additional File 1. A low GC content and a repetitive sequence in the methylation-variable transitional-CpG site often showed weak or smearing MSP bands due to the reduced complexity of the nucleotide sequences following bisulfite treatment [4,8,33]. In order to increase the specificity of transitional-CpG amplification, each MSP primer set was designed to contain 3-5 CpG sites and to encompass a small amplicon size, and the MSP reaction was conducted under stringent conditions with using dTTP-radioisotope as described previously [4,8,11,12]. Briefly, 10 μL of a PCR mixture that contained 1 μL bisulfite modified DNA, 1 μCi of α-^32^P dTTP (PerkinElmer, Boston, MA, USA), 62.5 μM dATP, dCTP and dGTP, 25 μM dTTP, 1 pmol of primers, 0.1% Tween 20 and 0.3 unit of *Taq *polymerase was amplified by 32 PCR cycles under hot-start PCR conditions. The representative methylation results are shown in Figure 1A.

**Table 1:** The nearest retroelement and the transcription of the 14 selected genes with and without CpG islands

Gene	CpG islands	Nearest retroelement		No. of expressed transcripts in the stomach
			
		Name of family	Distance to transcription start site	
*CDH1*	Yes	Alu	0.3 kb	19
*ARRDC4*	Yes	Alu	1.7 kb	7
*PPARG*	Yes	Alu	2.3 kb	3
*CDKN2A*	Yes	LTR	2.4 kb	1
*TRAPPC2L*	Yes	Alu	3.8 kb	14
*DUSP6*	Yes	Alu	6.6 kb	8
*MLH1*	Yes	Alu	6.6 kb	0
*RUNX3*	Yes	LTR	8.3 kb	0
*PGA5*	No	Alu	1.2 kb	734
*PGC*	No	Alu	1.6 kb	33
*TFF1*	No	Alu	0.5 kb	11
*TFF2*	No	L1	2.7 kb	632
*MSLN*	No	Alu	1.4 kb	50
*KRT6A*	No	Alu	4.2 kb	1

The information concerning CpG islands, retroelements and the number of expressed transcripts in the stomach was obtained from previously published data [4].

### Semiquantitative evaluation of transitional-CpG methylation variation

**Figure 2:**
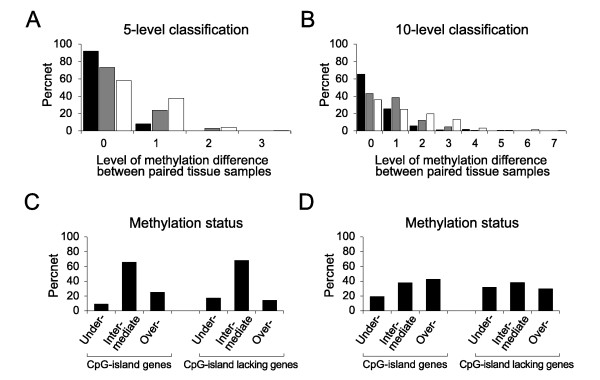
**Analysis of variable methylation in the transitional-CpG sites**. (A and B) The reproducibility of the 5-level classification with 20%-methylation increment (A) and 10-level classifications with 10%-methylation increment (B) was verified by the proportion of the paired tissue samples showing no differences in the level of methylation. The duplicated DNAs of the same tissue (closed bars), a pair of 1-cm-adjacent tissues (grey bars) and a pair of antrum and body tissues (open bars) were compared. (C and D) Analysis of an intermediate methylation level. The frequency of undermethylated and overmethylated cases was estimated based on an intermediate level spanning two levels (20% methylation) (C) and one level (10% methylation) (D) of 10-level classification common in *H. pylori*-negative gastric mucosa.

The PCR condition of each MSP primer set was validated by plotting the standard curve according to various mixtures of band intensity from the MSP products with universal methylated and unmethylated control DNA [11,18]. The genomic DNA treated with DNA methylase (CpGenome Universal Methylated DNA, Chemicon, Temecula, CA, USA) and amplified by a universal primer (5'-CCG ACT CGA GNN NNN NAT GTG G-3') were used for the universal methylated and unmethylated control DNA, respectively. Based on the standard curve, the methylation density of the transitional-CpG site was calculated using the following formula: methylation proportion (%) = (methylation intensity/(methylation + unmethylation intensity)) × 100 [8,11,13,15,18]. To validate the reproducibility of the variable methylation density using a semiquantitative analysis, the 5-level classification with 20%-methylation increment and the 10-level classification with 10%-methylation increment were compared in the paired tissue samples. The comparative analysis of the paired samples was conducted using the duplicated DNAs of the 40 tissues and 48 pairs of 1-cm-adjacent tissues in addition to 100 pairs of antrum and body tissues that were obtained from the 50 *H. pylori*-negative stomach tissues and the 50 *H. pylori*-positive stomach tissues (Figure 2).

**Figure 3:**
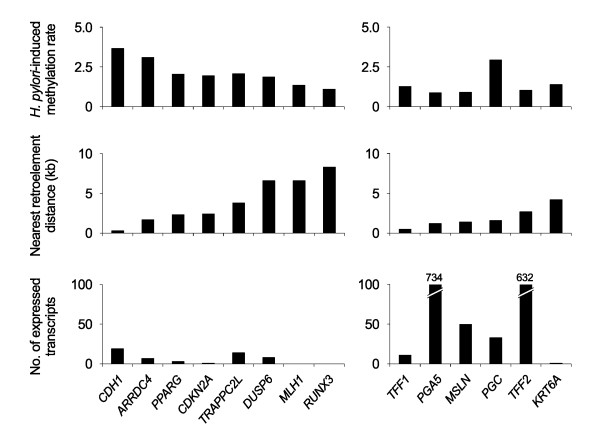
***H. pylori*-induced methylation of the CpG-island-containing and -lacking genes according to the distance to the nearest retroelements and the transcription activity**. A total of 14 transitional-CpG sites were grouped into eight CpG-island genes (*CDH1*, *ARRDC4*, *PPARG*, *CDKN2A*, *TRAPPC2L*, *DUSP6*, *MLH1 *and *RUNX3*) and six genes lacking CpG-islands (*PGA5*, *PGC*, *TFF1*, *TFF2*, *MSLN *and *KRT6A*). The gene-retroelement distance was estimated between the transcriptional start sites and the nearest Alu, L1 or LTR retroelements. The *H. pylori*-induced methylation rate was represented by the *H. pylori*-positive-to-negative ratio of the overmethylation rate. The overmethylation rate was calculated according to the relative proportion of overmethylated cases to the total sum of the over- and under-methylated cases. The transcriptional activity of each gene was shown with the number of expressed transcripts [4].

The overmethylation rate of each transitional-CpG site in the *H. pylori*-positive and -negative gastric mucosa was calculated by the relative proportion of overmethylated cases to the total sum of the over- and under-methylated cases. The *H. pylori*-associated overmethylation rate was calculated using the *H. pylori*-positive-to-negative ratio of each transitional-CpG-overmethylation rate. This was used to evaluate the relationship between the methylation of transitional-CpG sites and the distance between the transcription start site and the nearest retroelement (Figure 3).

### Cloning and sequencing of methylation-variable site

The MSP results of transitional-CpG sites were reconfirmed with cloning and sequencing of the common PCR primer sets encompassing both the unmethylated and methylated CpG sites (Figure 1B) [4,11,12]. The PCR products of the common primer sets were cloned into the T&A cloning vector (Real Biotech, Taipei, Taiwan). The DNA sequencing was performed for 10 clones of the common PCR vectors using the BigDye Termination Kit (PE Biosystems, Foster City, CA, USA) and an ABI automated DNA sequencer (PE Biosystmes, Warrington, UK). Because the common primer sets covering the CpG-poor regions produced various degrees of methylation according to the PCR condition, we adjusted the PCR condition of the common primer set to obtain the methylation density that was similar in both the semi-quantitative MSP assay and the sequencing results of the 10 common-PCR clones.

### Analysis of microsatellite alleles

For the PCR-based LOH analysis, a total of 40 microsatellite markers on eight cancer-associated chromosomes (3p, 4p, 5q, 8p, 9p, 13q, 17p and 18q) and the guidelines for scoring the status of LOH and MSI were used as described elsewhere [20,21]. The allelic profile of the 40 microsatellite sequences examined in each case was initially categorized into MSI based on the presence of novel alleles in the homozygous marker and the unilateral allelic loss in the heterozygous marker. According to the number of the LOH-positive chromosomes, the level of LOH was scored as low-level (two or three chromosomal losses, LOH-L) and high-level (four or more chromosomal losses, LOH-H) LOH. One or no chromosomal losses were classified into the baseline-level (LOH-B) for the diffuse-type cancer and into the low level for intestinal and mixed type, respectively, depending on their corresponding histological subtype.

### Statistical analysis

Fisher's exact test was used for comparing the over- and under-methylation changes between the *H. pylori*-negative or -positive gastric mucosa and the gastric cancers, with significance assigned to values below *P *values less than 0.01. The Student's *t *test was used for comparing the methylation differences between the gastric noncancerous and cancerous tissues, with significance assigned to values below *P *values less than 0.05. Statistical evaluation was performed by using the statistical software package SPSS 11.0 (SPSS Inc., Chicago, IL, USA).

## Results

### Methylation-variation in noncancerous tissues

A total of 14 transitional-CpG sites were chosen from eight CpG-island genes (*CDH1*, *ARRDC4*, *PPARG*, *CDKN2A*, *TRAPPC2L*, *DUSP6*, *MLH1 *and *RUNX3*) and six CpG-island-lacking genes (*PGA5*, *PGC*, *TFF1*, *TFF2*, *MSLN *and *KRT6A*) (Table 1). The reproducibility of transitional-CpG site amplified by each MSP primer set was evaluated with the duplicated DNAs of the same tissue, a pair of 1-cm-adjacent tissues and a pair of antrum and body tissues (Figure 2A and 2B). 92% of the 40 duplicated DNAs, 73% of the 48 1-cm-adjacent tissue pairs, and 58% of the 100 antrum and body pairs were scored as the same methylation levels in the 5-level classification. The same-scored cases of the duplicated experiments were approximately 25% more frequent in the 5-level classification than in the 10-level classification.

An intermediate level of variable transitional-CpG methylation was analyzed using 1,400 MSP amplicons of the 14 transitional-CpG sites obtained from 100 *H. pylori*-negative stomach samples (Figure 2C and 2D). In the 10-level classification, the fraction of a single common methylation level (38%) was so low that an intermediate level was determined based on two common levels. 937 (67%) of the 1,400 MSP amplicons were categorized into an intermediate level in the 100 tissues. Intermediate methylation levels of eight CpG-island margins tended to be lower than those of six CpG-island-lacking sites (Table 2). Overall, large fractions of individual stomach tissues (67%) and 1-cm-adjacent tissue pairs (73%) showed that the transitional-CpG sites were methylated in a range of similar densities varying between about 20%. Therefore, an intermediate level spanning two levels (20% methylation) of 10-level classification was used to determine the over- or under-methylated status of transitional-CpG sites in the gastric mucosa.

**Table 2:** The frequency of under- and over-methylated cases as determined based on intermediate methylation levels common in the *H. pylori*-negative gastric mucosa (n = 100)

Gene name	Intermediate methylation density	No. of under- methylated cases	No. of intermediate methylation cases	No. of over- methylated cases
CpG-island containing genes				
*CDH1*	0 - 20%	0	79	21
*ARRDC4*	0 - 20%	0	80	20
*PPARG*	0 - 20%	0	60	40
*CDKN2A*	0 - 20%	0	54	46
*TRAPPC2L*	40 - 60%	13	81	6
*DUSP6*	20 - 40%	26	62	12
*MLH1*	20 - 40%	20	61	19
*RUNX3*	30 - 50%	5	53	42

CpG-island lacking genes				
*PGA5*	40 - 60%	10	78	12
*PGC*	40 - 60%	29	67	4
*TFF1*	20 - 40%	11	52	37
*TFF2*	30 - 50%	36	60	4
*MSLN*	60 - 80%	0	88	12
*KRT6A*	60 - 80%	17	65	18

### Relationship between *H. pylori*-associated overmethylation either the retroelements or transcription activity

The frequencies of the over- and under-methylated cases were separately counted to estimate alterations in the variable methylation of the transitional-CpG sites (Table 3). On the analysis of the overmethylated cases, all the CpG-island margins were significantly overmethylated in the *H. pylori*-infected gastric mucosa and none of the CpG-island-lacking sites showed a significant difference for the frequency of the overmethylated cases. On the analysis of the undermethylated cases, the frequency of the undermethylated *TFF1 *gene was significantly low in the *H. pylori*-positive gastric mucosa (*P *= 0.003). The methylation data of bone marrow was cited from a previous study using the same radioisotope-labeling MSP protocol [4]. In bone marrow, the *PGC*, *TFF1*, *MSLN *and *RUNX3 *genes were completely methylated, whereas most of the CpG-island genes were completely unmethylated.

**Table 3:** Comparison of the frequency of over- and undermethylated genes detected in the gastric non-cancerous and cancerous tissues

Gene	Noncancerous tissue		Microsatellite genotype of gastric cancers (%)				Bone marrow
			
	*H. pylori *negative (n = 100)	*H. pylori *positive (n = 100)	LOH-B (n = 13)	LOH-L (n = 29)	LOH-H (n = 24)	MSI (n = 4)	(n = 18) (%)
Overmethylation frequency							
*CDH1*	21	77*	0 (0)*	7 (24)	3 (13)	0 (0)	0 (0)
*ARRDC4*	20	62*	3 (23)	8 (28)	1 (4)*	1 (25)	0 (0)
*PPARG*	40	82*	5 (38)	7 (24)	6 (25)	1 (25)	0 (0)
*CDKN2A*	46	90*	9 (69)	16 (55)	9 (38)	2 (50)	4 (22)
*TRAPPC2L*	6	21*	6 (46)*	8 (28)*	1 (4)	1 (25)	0 (0)
*DUSP6*	12	36*	3 (23)	3 (10)	1 (4)	2 (50)	0 (0)
*MLH1*	19	37*	4 (31)	17 (59)*	11 (46)	3 (75)	0 (0)
*RUNX3*	42	85*	10 (77)	26 (90)*	19 (79)*	4 (100)	18 (100)
*PGA5*	12	13	5 (38)	8 (28)	1 (4)	0 (0)	0 (0)
*PGC*	4	10	4 (31)	4 (14)	5 (21)	1 (25)	18 (100)
*TFF1*	37	52	5 (38)	3 (10)*	3 (13)*	0 (0)	18 (100)
*TFF2*	4	3	4 (31)	4 (14)	1 (4)	0 (0)	2 (11)
*MSLN*	12	11	8 (62)*	16 (55)*	6 (25)	1 (25)	18 (100)
*KRT6A*	18	16	4 (31)	16 (55)*	4 (17)	3 (75)	3 (17)

Undermethylation frequency							
*CDH1*	0	0	0 (0)	0 (0)	0 (0)	0 (0)	0 (0)
*ARRDC4*	0	0	0 (0)	0 (0)	0 (0)	0 (0)	0 (0)
*PPARG*	0	0	0 (0)	0 (0)	0 (0)	0 (0)	0 (0)
*CDKN2A*	0	0	0 (0)	0 (0)	0 (0)	0 (0)	0 (0)
*TRAPPC2L*	13	11	1 (8)	2 (7)	7 (29)	1 (25)	16 (89)
*DUSP6*	26	25	3 (23)	13 (45)	12 (50)	2 (50)	18 (100)
*MLH1*	20	18	7 (54)	6 (21)	9 (38)	1 (25)	17 (94)
*RUNX3*	5	1	0 (0)	1 (3)	2 (8)	0 (0)	0 (0)
*PGA5*	10	14	0 (0)	3 (10)	5 (21)	0 (0)	0 (0)
*PGC*	29	18	4 (31)	8 (28)	5 (21)	2 (50)	0 (0)
*TFF1*	11	1*	7 (54)	19 (66)*	17 (71)*	3 (75)	0 (0)
*TFF2*	36	26	4 (31)	11 (38)	8 (33)	3 (75)	13 (72)
*MSLN*	0	0	0 (0)	1 (3)	1 (4)	2 (50)	0 (0)
*KRT6A*	17	16	3 (23)	3 (10)	3 (13)	1 (25)	5 (28)

The gastric cancers were categorized into baseline-level LOH (LOH-B), low-level LOH (LOH-L), high-level LOH (LOH-H) and microsatellite instability (MSI). The details are described in the material and methods section.

*H. pylori-positive gastric mucosa and gastric cancers were compared with *H. pylori*-negative gastric mucosa. Significant differences were determined using Fisher's exact test (*P *< 0.01).

The relationship between the transitional-CpG methylation and the distance to the nearest retroelement was evaluated using the *H. pylori*-associated overmethylation rate (Figure 3). In the CpG-island genes, the *H. pylori*-associated overmethylation rate was higher as the distance of the nearest retroelement became shorter. The CpG-island-lacking genes that showed no significant methylation difference between the *H. pylori*-positive and -negative mucosa were methylated irrespective of the distance of the nearest retroelement.

**Figure 4:**
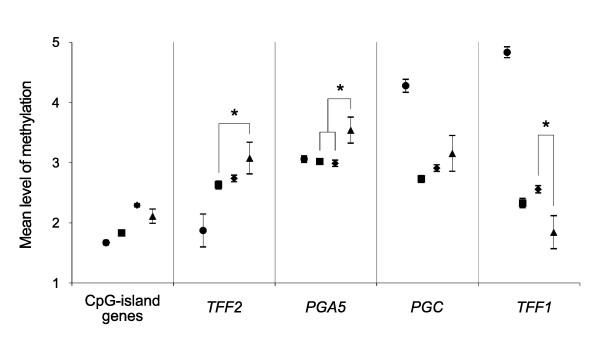
**The methylation in the bone marrow and the gastric noncancerous and cancerous tissues**. The mean level of methylation in the bone marrow (●), the *H. pylori*-negative gastric mucosa (■), the *H. pylori*-positive gastric mucosa (♦) and the baseline-level LOH cases (▲) was compared. Asterisks indicate significant differences between the noncancerous stomach tissues and the baseline-level LOH cancers (*P *< 0.05). Student's *t *test was used. Error bars indicate the standard error of the mean.

The methylation of transitional-CpG sites was compared between the stomach and bone marrow in terms of the transcription activity (Table 1 and Figure 4). The CpG-island gene group that was weakly active in the stomach was more methylated in the *H. pylori*-negative and -positive tissues than that in the bone marrow. Of the CpG-island-lacking *TFF2 *and *PGA5 *genes that were most highly expressed in the stomach, the mean methylation level of the *TFF2 *gene was lower than in the bone marrow (1.9) than in the *H. pylori*-negative (2.6, *P *= 0.015) and -positive mucosa (2.7, *P *= 0.015). The mean methylation level of the *PGA5 *gene was similar in the bone marrow (3.1) and *H. pylori*-negative (3.0) and -positive mucosa (3.0). The *PGC *and *TFF1 *stomach-specific genes, which were weakly active in the gastric mucosa when compared with the two master stomach-specific genes, were densely methylated in bone marrow (mean methylation level, 4.3 and 4.8).

### Methylation patterns in the gastric cancer under the influence of LOH

The gastric cancers examined by PCR-based LOH analysis were categorized into baseline-level LOH (LOH-B), low-level LOH (LOH-L), high-level LOH (LOH-H), and MSI as was reported previously [20,21]. Thirteen LOH-B (19%), 29 LOH-L (41%), 24 LOH-H (34%), and 4 MSI (6%) cases were identified from 70 gastric cancer samples. Overall, the frequency of both the overmethylated genes with and without CpG-islands was significantly lower in the LOH-H cases (21%) than that in LOH-L (35%, *P *< 0.0001) and LOH-B cases (38%, *P *< 0.0001). In comparison of the methylation levels in the noncancerous and LOH-B cancerous tissues, the CpG-island genes in the gastric cancers tended to be more methylated than those in the *H. pylori*-negative tissues and to be less methylated than those in the *H. pylori*-positive tissues (Figure 4). In analysis of the CpG-island-lacking genes, the mean methylation level of the *TFF2 *(3.1 vs. 2.6, *P *= 0.029) and *PGA5 *(3.5 vs. 3.0, *P *= 0.035) master genes was high in the LOH-B cases compared with that of the *H. pylori*-negative tissue. Meanwhile, the mean methylation level of the *TFF1 *gene was low in the LOH-B cases compared with the *H. pylori*-positive cases (1.8 vs. 2.6, *P *= 0.024).

**Figure 5:**
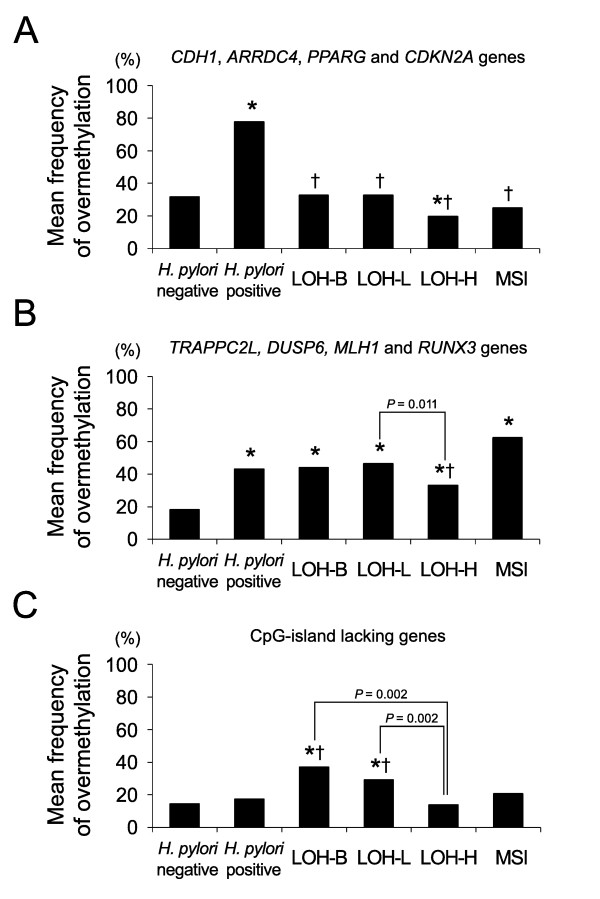
**The frequency of overmethylated cases was compared between the gastric noncancerous and cancerous tissues**. A total of 14 genes that were examined were grouped into three categories: (A) CpG-island-containing genes less than 3-kb close to the nearest retroelement; (B) CpG-island-containing genes more than 3-kb distant from the nearest retroelement; (C) The *PGA5*, *PGC*, *TFF1*, *TFF2*, *MSLN *and *KRT6A *genes. The asterisks and crosses indicate significant differences compared with the *H. pylori *negative and *H. pylori *positive gastric mucosa, respectively (*P *< 0.05). Student's *t *test was used.

On the analysis of four CpG-island genes located within a 3-kb distance to the nearest retroelements (*CDH1*, *ARRDC4*, *PPARG *and *CDKN2A*) (Figure 5A), the frequency of overmethylated genes was significantly low in the gastric cancers (LOH-B, 33%, *P *< 0.0001; LOH-L, 33%, *P *< 0.0001; LOH-H, 20%, *P *< 0.0001) when compared with the *H. pylori*-positive gastric mucosa (78%). The *TRAPPC2L*, *DUSP6*, *MLH1 *and *RUNX3 *genes more than 3-kb distal from the nearest retroelements were similarly overmethylated in the *H. pylori *positive mucosa (43%) and gastric cancers (LOH-B, 44%; LOH-L, 47%; LOH-H, 33%) (Figure 5B). On the analysis of the CpG-island-lacking gene group (Figure 5C), the overmethylated genes were significantly frequent in the LOH-B (37%, *P *= 0.015 and 0.006) and LOH-L (29%, *P *= 0.001 and < 0.0001) cases when compared with that of the *H. pylori*-positive (18%) and *H. pylori*-negative (15%) mucosa.

Of the 14 genes that were examined, the *TFF1 *gene closest to the retroelements was undermethylated in the gastric cancers irrespective of the LOH level (Figure 3 and Table 3). On the contrary, the *MHL1, RUNX3 *and *KRT6A *genes that showed few transcripts were far away from the retroelements and they were frequently overmethylated in the LOH-L cases (Table 1 and Table 3). The number of MSI cases detected in this study was not enough to be statistically analyzed for the methylation changes. However, the frequency of the overmethylated genes distant from the nearest retroelements was highest in the MSI cases (Table 3 and Figure 5B).

## Discussion

**Figure 6:**
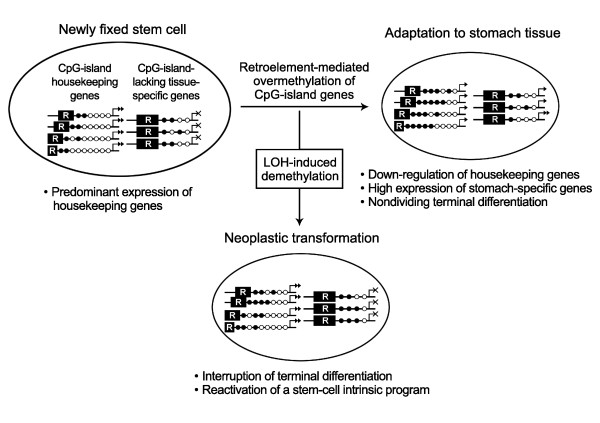
**Schematic diagram of the adaptive differentiation and neoplastic transformation of newly fixed stem cells**. In the newly fixed stem cells in the gastric mucosa, the gene-adjacent retroelements (R) drive the concurrent methylation of CpG-island genes in a distance-dependent manner. Given the interaction of the stomach-specific genes lacking CpG-islands and the housekeeping genes containing CpG-islands, which share the limited amount of nuclear proteins in a nuclear space, the overmethylation of CpG-islands down-regulates the housekeeping genes and up-regulate the stomach-specific genes. A high expression of stomach-specific genes promotes the non-dividing terminal differentiation of newly fixed stem cells in the stomach tissue-environment. The LOH events reducing a gene dose lead to 1) dose-compensatory demethylation, 2) the interruption of terminal differentiation, and 3) reactivation of a stem-cell intrinsic program for cell migration. Additionally, the highly expressed stomach-specific genes are overmethylated in a subset of gastric cancers and the overmethylated genes facilitate the interruption of terminal differentiation.

A comparative analysis of the whole transcripts between bone marrow and somatic tissues has illustrated that the CpG-island-containing and -lacking gene groups are expressed predominantly in pluripotent stem cells and somatic tissues inducing non-dividing terminal differentiation, respectively [4,12]. In particular, there is a great difference in the number of SAGE transcripts between the highly expressed master-specific gene group and weakly active housekeeping gene group in the stomach (Table 1) [4,12]. The *H. pylori*-infected gastric mucosa that promotes the recruitment of bone marrow stem cells has been associated with the overmethylation and down-regulation of CpG-island genes, which paves the way for the non-dividing terminal differentiation of the newly fixed stem cells [4]. In this study, all of the eight CpG-island genes and none of the six CpG-island-lacking genes were overmethylated in the *H. pylori*-infected mucosa (Table 3). This agrees with a previous study [4] reporting that the inverse relationship between the overmethylated CpG-island genes and the non-overmethylated stomach-specific genes lacking CpG-islands in a nuclear space facilitates the high expression of stomach-specific genes for the stomach-specific terminal differentiation of marrow-derived stem cells (Figure 6).

There are three major retroelements in the human genome, Alu, L1, and LTR, of which Alu copies are short in size and enriched in close proximity to the CpG-island genes (Table 1) [11]. The extent of CpG-islands is closely related to the distance between the host genes and the parasitic retroelements [11]. In this study, the CpG-island genes containing a short transitional-CpG segment close to the Alu elements were more frequently overmethylated than those containing a long transitional-CpG segment distant from the Alu elements in the *H. pylori*-positive gastric mucosa (Figure 3). A previous study on the trans-differentiation of marrow and adipose stem cells has also shown that the adipocyte differentiation of marrow stem cells results in the overmethylation of short transitional-CpG segments of the *PPARG *and *CDKN2A *genes but not long transitional-CpG segments of the *MLH1 *and *RUNX3 *genes [13]. It is likely that the gene-adjacent retroelements are associated with the concurrent overmethylation of numerous CpG-island genes in a distance-dependent manner, and this regulates the patterning of DNA methylation for the maintenance of both the highly expressed stomach-specific genes and the weakly expressed housekeeping genes. Therefore, the length of transitional-CpG sites is considered as a DNA methylation code that modulates the tissue-specific terminal-differentiation of marrow-derived stem cells [4].

Both the CpG-island-positive and -negative genes tended to be demethylated in a LOH-level-dependent manner, which was consistent with the previous reports [15,18]. The CpG-island genes in close proximity to the retroelements were undermethylated in gastric cancers when compared with that in the *H. pylori*-positive gastric mucosa, whereas the CpG-island genes distant from the retroelements were similarly overmethylated in the gastric cancers and *H. pylori*-positive mucosa (Figure 5). A previous study on marrow and adipose stem cells has also shown that short transitional-CpG segments are over- or undermethylated more dynamically than long transitional-CpG segments in response to trans-differentiation induction as well as H_2_O_2 _treatment [13]. Short transitional-CpG segments close to the retroelements that are readily overmethylated in the *H. pylori*-positive mucosa appear to be promptly demethylated with the LOH events (Figure 6).

When considering that the nuclear proteins binding to a gene-control region prevent the methylation spreading of parasitic retroelements [34], the transitional-CpG sites reflect a balance between the methylation spreading and transcriptional activity. In cancer tissue that undergoes the LOH event that reduces a gene dose, the remaining gene copies have the increased possibility of using the nuclear proteins and this would lead to the demethylation of transitional-CpG segments [3,4,12]. Previous studies have described the undermethylation and up-regulation of housekeeping genes in various types of invasive and migratory cells and tissues, such as embryo implantation, invasive placentation, stem cell migration, and cancer progression [4,35]. The dose-compensatory demethylation in gastric cancer appears to facilitate the interruption of non-dividing terminal-differentiation of newly fixed stem cells and to reactivates a stem-cell intrinsic program for cell migration and proliferation (Figure 6) [36].

Of the four CpG-island-lacking stomach-specific genes, *TFF2*, *PGA5*, *PGC *and *TFF1*, the *TFF2 *gene was undermethylated in bone marrow when compared with that of the gastric mucosa (Figure 4). The two stomach-specific genes (*PGA5 *and *TFF2*) were most highly expressed in the gastric mucosa (Table 1). The *PGA5 *gene was similarly methylated in bone marrow and gastric mucosa. The expression of the *PGC *and *TFF1 *genes that were densely methylated in bone marrow was relatively weak in the gastric mucosa (Table 1). Assuming the fixation of bone marrow derived stem cells in the gastric mucosa, the expression pattern of the four stomach-specific genes was consistent with their methylation patterns in bone marrow. According to a self-organization model [2], the stomach tissue-environment induces the high expression of master genes nucleating a transcription hub that recruits other many genes and nuclear proteins for gastric cell proliferation and differentiation (Figure 6). The methylation pattern of the four genes was similar in the *H. pylori*-positive and -negative gastric mucosa (Figure 4), and this implicates the coordinate expression of stomach-specific genes that initiate the adaptation of marrow-derived stem cells to the stomach tissue environment.

Even though the master stomach-specific genes and retroelements sustain the high-fidelity replication of concurrent methylation patterns, erroneous DNA methylation that is more common than erroneous DNA base sequences often gives rise to the aberrantly methylated genes [37,38]. The *TFF2 *and *PGA5 *master genes were found to be densely methylated in the LOH-B cases (Figure 4). The LOH-B cases having a few LOH events are likely to preserve the methylation pattern formed in cancer progenitor cells. In addition, the *TFF1 *gene in close proximity to the retroelement was undermethylated in gastric cancers (Table 3). This discordant methylation pattern of the stomach-specific genes may be improper to maintain a transcription hub in the stomach tissue-environment and to induce the non-dividing terminal differentiation of newly fixed stem cells (Figure 6).

Comparison of the *H. pylori*-positive and -negative gastric cancer tissue was not conducted, because the methylation status of the CpG-islands in the gastric cancerous and precancerous lesions has been found to be similar in the *H. pylori*-infected and non-infected patients [7]. In this study, the transitional-CpG sites in diffuse-type gastric cancers tended to be overmethylated in the LOH-B cases and to be undermethylated in the LOH-H genotype cases depending on the level of chromosomal losses. Additionally, both the over- and under-methylation frequencies of individual and overall transitional-CpG sites were not significantly different between the distinct histologic types of the LOH-H cases (data not shown). When assuming that the methylation of transitional-CpG sites promotes cell differentiation, the LOH-induced demethylation may lead to the dedifferentiation of gastric cancer cells. Therefore, the dynamic methylation pattern of transitional-CpG sites in a given gastric cancer appears to be determined according to the level of LOH rather than *H. pylori *infection and the differentiation state of cancer progenitor cells.

The transitional-CpG sites containing repeat sequences with a low CpG content often limit the use of an adequate MSP primer set. The real-time PCR protocol, which has been adapted for the quantitative estimation of CpG-island methylation, shows weak or non-specific signals when amplifying the transitional-CpG sites [4]. Diverse experimental protocols have demonstrated that the results of PCR-based methylation analyses are variable between experimental samples, and the analyses fluctuate according to the PCR conditions even with examining the same tissue DNA [39] (Figure 2A and 2B). Especially, a high number of amplification cycles is prone to biased expansion of the methylation amplicon in the analysis of small biopsy tissues. The radioisotope-labeling PCR with a minimal number of amplification cycles is necessary for the PCR-DNA-band's sharpness and reproducibility. In this study, to ensure reliable experimental results, an intermediate level of methylation variation was determined based on the reproducibility and variation range of the MSP density, as was estimated using the duplicated same tissue and pairs of adjacent tissues and the antrum and body tissues (Figure 2).

## Conclusions

According to the self-organization and dosage compensation models, several stomach-specific genes lacking CpG-islands and numerous housekeeping genes containing CpG-islands coordinate to establish gastric cell phenotypes in the newly fixed stem cells. This study suggests that the concurrent methylation of multiple CpG-island genes is initiated under the influence of nearby retroelement methylation in the gastric mucosa infected with *H. pylori*, and the LOH-induced DNA demethylation and discordant methylation of stomach-specific genes may facilitate gastric carcinogenesis by reactivating a stem-cell intrinsic program.

## Competing interests

The authors declare that they have no competing interests.

## Authors' contributions

SJH conceptualized, helped with data collection and analysis, and drafted the manuscript. JHO and EJJ helped with performing the endoscopic biopsy and data collection. KOM helped with performing the histopathologic examination. MIK and SWC conceived the study, participated in its design and contributed to the manuscript. MGR conceptualized, edited the manuscript for important intellectual content and has read and approved the final version of the manuscript. All authors read and approved the final manuscript.

## Pre-publication history

The pre-publication history for this paper can be accessed here:

http://www.biomedcentral.com/1471-230X/10/137/prepub

## Supplementary Material

Additional file 1 **List of MSP primer sets for the 14 transitional-CpG sites**. We summarized the MSP sites, sequences, and conditions for the 14 transitional-CpG sites. Click here for file
