# Supplementary material for: The overmethylated genes in Helicobacter pylori-infected gastric mucosa are demethylated in gastric cancers
Source: BMC Gastroenterol. 2010 Nov 20;10:137. doi: 10.1186/1471-230X-10-137 (PMC2995475; doi:10.1186/1471-230X-10-137)
Supplement: Additional file 1 — List of MSP primer sets for the 14 transitional-CpG sites. We summarized the MSP sites, sequences, and conditions for the 14 transitional-CpG sites. [file 1471-230X-10-137-S1.DOC]

**Additional file 1-** List of MSP primer sets for the 14 transitional-CpG sites

| **Gene** | **Genomic position** | |  | **Sequence of forward primer** | **Sequence of reverse primer** | **Size**  **(bp)** | **Tm**  **(^o^C)** |
| --- | --- | --- | --- | --- | --- | --- | --- |
| *CDH1* |  | 0 kb | U | GGTGAATTTTTAGTTAATTAGTGGTAT | TCACAAATACTTTACAATTCCAACA | 108 | 56 |
|  |  |  | M | TGAATTTTTAGTTAATTAGCGGTAC | ACAAATACTTTACAATTCCGACG | 104 | 58 |
| *ARRDC4* | Upstream | 0.3 kb | U | GAATGGTTGATATGTTGAGAGTT | CCCTAACCAATAATAAAACAATACA | 112 | 55 |
|  |  |  | M | GAACGGTTGATACGTTGAGAGTC | CCCTAACCAATAATAAAACGATACG | 112 | 53 |
| *PPARG* | Upstream | 1.5 kb | U | AGAAGAGAAAATTAAGGGATTT | ATAACTTACCCTTCACACAACA | 117 | 58 |
|  |  |  | M | AGAAGAGAAAATTAAGGGATTC | ATAACTTACCCTTCACACGACG | 117 | 58 |
| *CDKN2A* | Upstream | 1.6 kb | U | TTGGGATTAGGTTTAGTTTTGG | CTATAAAACCCTATCAACTCACACT | 130 | 58 |
|  |  |  | M | TCGGGATTAGGTTTAGTTTCG | AAACCCTATCGACTCACGCT | 125 | 60 |
| *TRAPPC2L* | Upstream | 0.7 kb | U | GGGAGTGATTTTTAGGGTGAT | TCCCCCATCTAACCTTTCCCA | 129 | 53 |
|  |  |  | M | GGGAGTGATTTTTAGGGCGAC | TCCCCCGTCTAACCTTTCCCG | 129 | 53 |
| *DUSP6* | Upstream | 2.1 kb | U | GTTTTAGTTGGGGGAGGTTTGT | TCCCAATAACTAAAACATCTCA | 100 | 56 |
|  |  |  | M | GTTTTAGTCGGGGGAGGTTTGC | TCCCAATAACTAAAACGTCTCG | 100 | 56 |
| *MLH1* | Upstream | 1.0 kb | U | GATTTTAGGATTGTTGATATGAGT | AAACTACCTCCTAATCTTTATCCA | 126 | 58 |
|  |  |  | M | GATTTTAGGATTGTCGATATGAGC | AACTACCTCCTAATCTTTATCCG | 125 | 58 |
| *RUNX3* | Upstream | 1.7 kb | U | TGGGGTTAGATTTTTGTTGTTTTT | ATAAAATCTTACAACCACCATCA | 107 | 56 |
|  |  |  | M | CGGGGTTAGATTTTCGTTGTTTTC | ATAAAATCTTACGACCACCGTCG | 107 | 58 |
| *PGA5* |  | 0 kb | U | GATTTTTAGAGGTTGATAAGGT | TTCTTCCCAAATCCCAACTCA | 115 | 55 |
|  |  |  | M | GATTTTTAGAGGTCGATAAGGC | TTCTTCCCGAATCCCAACTCG | 115 | 55 |
| *PGC* | Upstream | 0.2 kb | U | GGTGTATTTTGTGTTTTGTGTATT | ACTTACACCTCCTAACCTCCA | 117 | 60 |
|  |  |  | M | GTGTATTTTGTGTTTCGTGTATC | GCTTACACCTCCTAACCTCCG | 118 | 60 |
| *TFF1* | Downstream | 0.1 kb | U | TGGGTTTTGGTTAGGGTGTT | CTCATCCCTAACTCAAAATCA | 123 | 56 |
|  |  |  | M | TGGGTTTCGGTTAGGGTGTC | CTCATCCCTAACTCGAAATCG | 123 | 56 |
| *TFF2* | Upstream | 0.2 kb | U | GGTAGTTGTGTTTTGTGTAGGT | CACATAACCAATTTTCCACA | 130 | 56 |
|  |  |  | M | GGTAGTTGTGTTTTGTGTAGGC | CACGTAACCGATTTTCCACG | 130 | 62 |
| *MSLN* | Upstream | 0.8 kb | U | GGAGAGATTAGAGATGATTGTTGT | CATAAACTCTTATCCCCAATACA | 103 | 55 |
|  |  |  | M | GGAGAGATTAGAGATGATCGTCGC | CGTAAACTCTTATCCCCAATACG | 103 | 60 |
| *KRT6A* | Downstream | 0.1 kb | U | TTTTGGAGTGGGATATGGAGAT | AAAACCACAACAACAACCACCA | 104 | 60 |
|  |  |  | M | TTTTGGAGCGGGATACGGAGAC | AAAACCACAACAACAACCGCCG | 104 | 60 |

ABBREVIATION. U, unmethylation primer; M, methylation primer.
